# Sociodemographic Factors Associated with the Level of Knowledge of Early Postpartum Women about Oral Health Prevention in Infants Aged 0 to 2 Years Old: A Cross-Sectional Study under a Multivariable Analysis

**DOI:** 10.3390/ijerph20031881

**Published:** 2023-01-19

**Authors:** Nilda Gaspar-Damaso, Marysela Ladera-Castañeda, Nancy Córdova-Limaylla, Gissela Briceño-Vergel, Luis Cervantes-Ganoza, Miriam Nicho-Valladares, Alberto Cornejo-Pinto, Alí Echavarría-Gálvez, César Cayo-Rojas

**Affiliations:** 1 School of Stomatology, Universidad Privada San Juan Bautista, Lima 15067, Peru; 2 Grupo de Investigación Salud y Bienestar Global, Faculty of Dentistry and Postgraduate School, Universidad Nacional Federico Villarreal, Lima 15001, Peru; 3 Faculty of Stomatology, Universidad Inca Garcilaso de la Vega, Lima 15084, Peru

**Keywords:** knowledge, dentistry, early postpartum women, factors associated, oral health

## Abstract

The objective was to evaluate the sociodemographic factors associated with the level of knowledge of early puerperal women about oral health prevention in infants. This cross-sectional and analytical study evaluated 303 early puerperal women from a hospital in the Peruvian capital. A validated 18-question questionnaire was used to measure the level of knowledge. A logistic regression model was used to evaluate the influence of age, marital status, educational level, number of children, monthly income, and having a dentist as a family member. A significance of *p* < 0.05 was considered. A total of 46.86%, 30.36%, and 22.77% of the puerperal had poor, fair, and good knowledge, respectively. The risk of having poor knowledge was two times higher (OR = 2.43; CI: 1.26–4.70) in early postpartum women aged 18 to 25 years than in those older than 35 years. Early postpartum women with no education, primary and secondary education were 11 times (OR = 11.76; CI: 2.41–57.43), 6 times (OR = 6.61; CI: 1.72–25.45), and 5 times (OR = 5.50; CI: 1.52–19.89), respectively, more likely to have significantly poor knowledge compared to those with university education. In conclusion, only a small minority of early postpartum women had a good knowledge of oral health prevention in infants aged 0 to 2 years. Younger and less educated puerperal were at greater risk of having little knowledge on this topic. Finally, not having basic education was the main risk factor identified.

## 1. Introduction

Oral diseases constitute a public health problem because they can negatively affect people throughout their growth and development, causing pain, discomfort, loss of function, and reducing their quality of life [1,2]. These diseases affect approximately 3.5 billion people worldwide. It has also been reported that dental caries in permanent teeth is the most frequent disorder, and it is estimated that approximately 2000 million people suffer from it. On the other hand, it has been reported that 514 million children worldwide suffer from caries in deciduous teeth [3]. In Peru, the prevalence of carious lesions in enamel and dentin was reported to be 91.2% in 3-year-old children living in Lima [4].

The postpartum period, also known as puerperium, is a stage of maternal anatomical and physiological changes that begins with the production of breast milk and the physiological recovery of various organs and systems. This period is divided into three phases: Acute or immediate phase that occurs up to the first 24 h postpartum, intermediate or early phase from the 2nd to the 7th postpartum day, and late or remote phase up to 6 weeks postpartum to 6 months [5]. The puerperium or postpartum period is a key moment for health education since women are interested in information about the care and well-being of their newborns. It is therefore important to provide them with basic notions about preventive measures such as the recommended frequency of brushing, correct use of fluoride toothpaste, use of pacifiers, improper use of bottles, breastfeeding, and type of feeding, among others [6,7,8,9].

In this regard, the World Health Organization [3] has considered it essential to work on the prevention and control of oral diseases with specific actions during the preconception, prenatal and postnatal periods, implementing the promotion of self-care, community actions, and preventive activities due to the vulnerability of newborns [10,11]. Therefore, it is important to consider gestation, puerperium, and the first two years of life as stages of opportunities to promote health and foster healthy growth and development. To achieve this, the mother is a key factor in guiding good health behavior if she acquires good oral hygiene practices and healthy eating habits. This would make it possible to maintain oral health throughout the life cycle, since habits acquired at an early age are more likely to be preserved uninterrupted into adulthood [10,12,13,14]. However, the modulation of these habits can be influenced by some factors such as the level of knowledge about oral health, age, socioeconomic status, or educational level, among others [2,3,15,16].

Several studies have been conducted to determine the level of knowledge of pregnant women in order to adopt governmental policies and carry out educational interventions to improve their quality of life and that of their future babies, taking into account that both are considered vulnerable populations due to their greater exposure to risk factors [8,11,17,18]. However, in the last five years (2018 to 2022) there is only one study that evaluates the knowledge of early postpartum women about oral health in infants [19].

Therefore, the present study aimed to evaluate the sociodemographic factors associated with the level of knowledge of early postpartum women about oral health prevention in infants aged 0 to 2 years. The present study considers as a null hypothesis that there are no sociodemographic factors significantly associated with the level of knowledge of early postpartum women about oral health prevention in infants aged 0 to 2 years.

## 2. Materials and Methods

### 2.1. Study Design

The present observational, prospective, cross-sectional, and analytical study was conducted in early postpartum women attended at the Gynecology Service of the Carlos Lanfranco La Hoz Hospital of Puente Piedra, Lima, Peru; during the months of January to April 2022, and was written according to the STrengthening the Reporting of OBservational studies in Epidemiology (STROBE) guidelines [20].

### 2.2. Population and Selection of Participants

The sample size was *n* = 303 early postpartum women. This was calculated with the statistical program Epidat 4.2 using a formula to estimate a proportion from a population of N = 1244 early postpartum women (taking as reference the total care of these patients in the year 2021) considering a significance level α = 0.05, an estimated error of 5% and an expected proportion *p* = 0.5 (to obtain the largest possible sample size). The sampling method was systematic random with a 4 by 4 (N/n) selection bootstrap to complete the sample size and taking into consideration the following eligibility criteria:

#### 2.2.1. Inclusion Criteria

- Early postpartum women.
- Early postpartum women who gave voluntary informed consent.
- Early postpartum women of legal age (≥18 years).
- Early postpartum women without serious complications.

#### 2.2.2. Exclusion Criteria

- Early postpartum women who did not complete the questionnaire.

### 2.3. Variables

The response variable considered in the present study was the level of knowledge about oral health prevention in infants aged 0 to 2 years old. The independent variables were: Age [19,21,22], marital status, educational level [18,21], number of children, monthly family income [21], and having a dentist as a family member [21].

### 2.4. Instrument Design, Validation, and Application

An 18-question questionnaire [23] was improved based on the literature available in databases such as Scopus and Pubmed. Subsequently, the pertinence, objectivity, relevance, timeliness, and clarity of the questionnaire content were validated by three experts in dental public health and research, obtaining an acceptable Aiken’s V (V = 0.94, CI: 0.90–0.96) (See Supplementary Materials).

To evaluate the internal consistency of the questionnaire, the Kuder Richardson test (KR-20) was used, obtaining a value of 0.76, which is considered acceptable. To evaluate the reproducibility of the questionnaire, 30 randomly selected early postpartum women were surveyed over a period of 10 days, at two different times, and altering the order of the questions to avoid memory bias [24]. The intraclass correlation coefficient (ICC) of the scores obtained was acceptable (ICC = 0.97; 95% CI: 0.95–0.99).

### 2.5. Procedure

After obtaining permission from the Director of the Carlos Lanfranco La Hoz Hospital in Puente Piedra, the questionnaire was distributed personally to the gynecology service in a heteroadministered form. Before the early postpartum women answered the questions, any doubts about the objective of the work and the risk/benefit were clarified. It was also stressed to them that they could withdraw from the study at any time if they felt any discomfort in answering the questions. The first sheet of the questionnaire contained the informed consent form. Only the principal investigator collected the information and tabulated the data in a Microsoft Excel 2019 spreadsheet. All researchers had access to this information, which was stored on a digital handheld device with a password to maintain confidentiality. All printed information was destroyed for security. At the end of the study, the results were sent to those early postpartum women who requested the information from the principal investigator by e-mail.

### 2.6. Statistical Analysis

The Statistical Package for the Social Sciences (SPSS) version 28.0 was used for data analysis. For descriptive statistics, the results were presented with absolute and relative frequencies for qualitative variables. Measures of central tendency (mean) and dispersion (standard deviation) were used for quantitative variables. To estimate the level of general knowledge (poor, fair, and good) and its three dimensions (feeding habits, oral hygiene, and dental care), the Stanones scale [mean ± 0.75 (standard deviation)] was used for the cut-off points. For the bivariate analysis, the Pearson chi-square test with Yates correction for expected values less than 5 was applied. For the multivariate analysis, the risk factors were evaluated using a logistic regression model (logit model) with an odds ratio (OR), considering a significance of *p* < 0.05.

### 2.7. Bioethical Considerations

The present study was approved by an Institutional Research Ethics Committee of the Universidad Privada San Juan Bautista with resolution No. 1579-2021-CIEI-UPSJB, in accordance with the bioethical principles of freedom, confidentiality, non-maleficence, and respect for medical research on human beings, as set forth in the Declaration of Helsinki [25].

## 3. Results

### 3.1. Description of Sample

The present study included a total of 317 participants. However, 14 early postpartum women did not complete the questionnaire. The mean age of the 303 early postpartum women belonging to the gynecology service of a Peruvian public hospital was 28.1 ± 6.4 years. The predominant age group was 18 to 25 years (41.3%). Of the early postpartum women, 83.8% were married or cohabiting, 41.9% had studied only up to secondary school and 47.5% had two children. Regarding monthly family income, 46.2% of the respondents earned between US$125 and US$250 per month. Finally, 88.8% of the early postpartum women reported not having a dentist as a family member. (Table 1).

### 3.2. Analysis of the Principal Components of the Questionnaire

Regarding the questionnaire, the principal component factor analysis with Varimax rotation (in a pilot study with 180 participants) indicated three dimensions distributed in: D1 (knowledge about feeding habits in infants aged 0 to 2 years) (Q1–Q6), D2 (knowledge about oral hygiene in infants aged 0 to 2 years) (Q7–Q12) and D3 (knowledge about dental care in infants aged 0 to 2 years) (Q13–Q18). The correlation determinant was 0.034 and the Kaiser-Mayer-Olkin and Bartlett’s sphericity tests indicated a value of 0.653 and *p* < 0.001; respectively. All values were considered acceptable.

### 3.3. Knowledge about Feeding Habits

Regarding the knowledge of early postpartum women about feeding habits in infants aged 0 to 2 years of age, statistically significant associations were obtained with Q2 and Q5 (*p* < 0.001 and *p* = 0.040; respectively). Educational level was significantly associated with Q1 (*p* < 0.001), Q2 (*p* = 0.003), Q3 (*p* = 0.005) and Q5 (*p* < 0.001). The number of children was significantly associated with Q3 (*p* = 0.038). Monthly family income was significantly associated with Q1 (*p* < 0.001), Q2 (*p* < 0.001), Q3 (*p* = 0.003) and Q5 (*p* < 0.001). Finally, having a dentist as a family member was significantly associated with Q2 (*p* = 0.006) and Q6 (*p* = 0.035). (Table 2).

### 3.4. Knowledge about Oral Hygiene

Regarding the knowledge of early postpartum women about oral hygiene in infants aged 0 to 2 years, statistically significant associations were obtained between the age group with Q8 and Q12 (*p* = 0.003, *p* = 0.002, and *p* = 0.015; respectively). Marital status was significantly associated with Q9 and Q11 (*p* = 0.004 and *p* = 0.010, respectively). Educational level was significantly associated with Q8 (*p* < 0.001), Q9 (*p* = 0.003), Q11 (*p* < 0.001) and Q12 (*p* = 0.003). Number of children was significantly associated with Q8, Q10 and Q12 (*p* = 0.002, *p* = 0.016 and *p* = 0.009, respectively). Finally, monthly family income was significantly associated with Q8 (*p* = 0.032) and Q11 (*p* < 0.001) (Table 3).

### 3.5. Knowledge about Dental Care

Regarding the knowledge of early postpartum women about dental care in infants aged 0 to 2 years, statistically significant associations were obtained between the age group and Q17 (*p* = 0.033). Marital status was significantly associated with Q13, Q15, Q16 and Q18 (*p* = 0.038, *p* = 0.030, *p* = 0.005 and *p* = 0.003, respectively). Educational level was significantly associated with Q13 (*p* < 0.001) and Q18 (*p* < 0.001). Monthly family income was significantly associated with Q13, Q14, Q15 and Q18 (*p* < 0.001, *p* = 0.031, *p* = 0.018 and *p* = 0.005, respectively). Finally, having a dentist as a family member was significantly associated with Q13 (*p* = 0.022) and Q15 (*p* = 0.042) (Table 4).

### 3.6. Overall Knowledge about Oral Health Prevention in Infants Aged 0 to 2 Years Old

**Figure 1:**
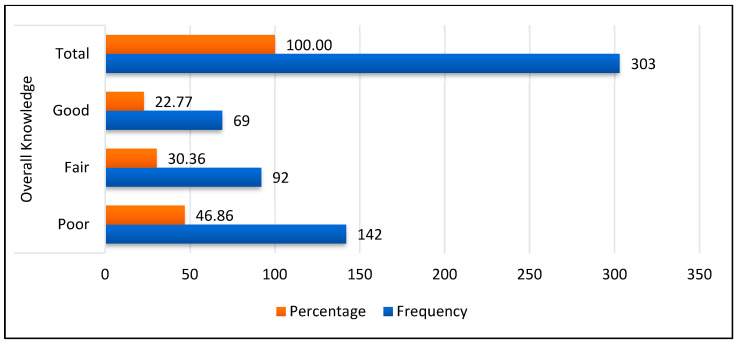
Frequency of early postpartum women’s overall knowledge about oral health prevention in infants aged 0 to 2 years old.

The 46.86% (CI: 41.24–52.48%) of the surveyed early postpartum women had poor knowledge about oral health prevention in infants aged 0 to 2 years old, while 30.36% (CI: 25.18–35.54%) and 22.77% (CI: 18.05–27.49%) showed fair and good knowledge, respectively (Figure 1).

### 3.7. Association of Sociodemographic Factors with Knowledge about Oral Health Prevention in Infants

On the other hand, it was observed that knowledge about eating habits in infants aged 0 to 2 years of age was significantly associated with age group (*p* = 0.007), educational level (*p* < 0.001) and monthly family income (*p* < 0.001). Knowledge about oral hygiene in infants aged 0 to 2 years was significantly associated with age group (*p* = 0.029), marital status (*p* = 0.005), educational level (*p* = 0.001), number of children (*p* = 0.005) and monthly family income (*p* = 0.020). In addition, knowledge about dental care in infants aged 0 to 2 years was significantly associated with age group (*p* = 0.003), educational level (*p* = 0.004), monthly family income (*p* = 0.012) and having a dentist as a family member (*p* = 0.019). Finally, overall knowledge about oral health prevention in infants aged 0 to 2 years was significantly associated with age group, marital status, educational level, and monthly family income (*p* < 0.001, *p* = 0.002, *p* < 0.001 and *p* < 0.001, respectively) (Table 5).

In the logistic regression analysis (logit model) of the crude model, the dependent variable was knowledge about oral health prevention in infants aged 0 to 2 years, the independent variable was age group and the possible confounding variables were marital status, educational level, number of children, monthly family income and having a dentist as a family member. As a result, the age group and educational level of the early postpartum women were found to be influential factors (*p* < 0.05). Subsequently, the adjusted model showed that early postpartum women between 18 and 25 years of age were significantly (*p* = 0.008) 2 times more likely to have poor knowledge (OR = 2.43; CI: 1.26–4.70) than those over 35 years of age. In addition, early postpartum women who had no education, those who only studied up to primary school, and those who only studied up to secondary school had 11 times (OR = 11.76; CI: 2.41–57.43), 6 times (OR = 6.61; CI: 1.72–25.45) and 5 times (OR = 5.50; CI: 1.52–19.89) respectively, the probability of having poor knowledge significantly (*p* < 0.05) compared to early postpartum women with university education (Table 6).

## 4. Discussion

The high prevalence of oral diseases [26] makes it necessary to promote and prevent oral health from early childhood to reduce the impact on future generations. This becomes even more relevant if we take into account that women during the gestation and postpartum stages are more predisposed to make changes in their habits, assimilating more easily the information that can benefit their health and that of their babies. Therefore, these stages become crucial to instruct mothers about oral health care so that they can be transmitters of healthy attitudes and practices to their children [8,9,26]. In Peru, 12.6 out of every 100 adolescents are pregnant or are already mothers according to the Demographic and Family Health Survey in 2018 [27]. Adolescents between 12 and 17 years of age represent 10.8% of the population in Peru. Of this total, adolescent pregnancies registered 10.1% in urban areas and 22.7% in rural areas [27]. Likewise, between 34% and 43% of patients in public hospitals have been reported with inadequate health literacy, being age, level of education, and unemployment are some of the factors that influence this problem [28]. For this reason, the National Concerted Health Plan 2007–2020 in Peru included as part of its health objective the inclusion of pregnant women in preventive dental care [29]. In view of the above, the present study aimed to determine the factors associated with the level of knowledge of early postpartum women about oral health prevention in infants aged 0 to 2 years old. The null hypothesis was rejected since academic level and age were identified as risk factors for having a poor level of knowledge about this topic. Taking into account the findings, it would be advisable for health authorities to pay more attention to this vulnerable population, focusing specifically on young women with low academic levels, encouraging their self-care and helping them to be guides for their children in the acquisition of healthy habits [7]. Furthermore, taking into account that maternal oral health is recognized as a risk factor for early childhood caries, preventive oral care, and educational sessions should be addressed before pregnancy and as part of prenatal and postnatal check-ups [30,31,32].

Of the total number of respondents, 48.86% had a poor level of knowledge. These results differ from those reported by Luengo et al. [17] who found that 58% of the women surveyed showed fair knowledge. This discrepancy could be due to the fact that the aforementioned study was conducted on Mexican pregnant women hospitalized in a specialized women’s health care facility where there is probably greater control and dedication to pre- and postnatal care, including oral health care. The present study was carried out in a general hospital where patients referred from different health facilities are attended. Likewise, the study by Luengo et al. was carried out before the COVID-19 pandemic, unlike the present study which was conducted when Peru was in the second wave of the pandemic and may have greatly limited the oral health care of early postpartum women. In addition, the poor level of knowledge obtained in the present study could be attributed to the low awareness of many women about their oral health, since despite having symptoms or signs of disease, they do not seek or receive promotional preventive dental care. If we add to this the socioeconomic level, it becomes a barrier to disease prevention [17,30,33,34]. Another explanation for this could be that oral health care instruction for the future baby is not routinely addressed during the mother’s prenatal care either because dentists are not part of the prenatal multidisciplinary health team or because there is little interprofessional education [30]. Furthermore, the present study was conducted in a population whose social determinants such as age and educational level may have had a negative influence on the level of knowledge [35,36,37].

In the present study, early postpartum women aged 18 to 25 years were significantly two times more likely to have poor knowledge than those over 35 years, which is consistent with the study by Ben David et al. [19] who reported that older women had greater knowledge. Likewise, Esteves et al. [22] found that women older than 30 years had acceptable knowledge in relation to knowing when their child should visit the dentist for the first time. Likewise, our results were similar to those found by Al-Sane et al. [21] who indicated that the age of the mother was significantly associated with a better level of knowledge about dental caries, since mothers older than 35 years had significantly better scores than younger mothers. In summary, these results could be explained by the fact that older women may want to become more skilled and acquire more experience, which may contribute to a healthier prenatal and postnatal environment [38]. Moreover, greater maturity would allow them to engage in independent, productive, and self-serving behaviors [39].

The early postpartum mothers in the present study who had no education, those who had only primary school, and those who had only secondary school had 11 times, 6 times, and 5 times, respectively, the probability of having poor knowledge significantly higher than those who had a university education. These results are similar to those obtained by Al-Sane et al. [21], who indicated that mothers with higher levels of education had better levels of knowledge. Furthermore, according to Barbieri et al. [18], a level of schooling equal to or higher than 8 years was associated with adequate knowledge about oral health. This could be explained by the fact that mothers with a higher educational level may have better discernment to choose the appropriate sources of information to adopt healthy practices. Likewise, women with a higher educational level may have better oral health literacy and a greater ability to choose a health service that is appropriate to their needs [40,41,42].

Among the limitations of the present study, we can mention that it was not possible to make a comparison between the knowledge of early postpartum women who attended in public health facilities and those who attended in private health facilities. Likewise, it was not possible to compare the knowledge of early postpartum women in urban areas with those in rural areas. Another limitation was the inability to interview early postpartum women under 18 years of age because informed consent could not be obtained from their guardians. Finally, the cross-sectional design of the present study did not allow us to evaluate the variation and durability of knowledge over time.

Taking into account the results obtained, it is recommended that health authorities develop oral healthcare programs focused on the mother-child duo from the moment of conception [10]. Likewise, it is necessary to empower professionals who work with pregnant women and postpartum women, whether primiparous or multiparous, on the need to develop promotion and prevention strategies during prenatal and postnatal control in an interdisciplinary manner with the dentist, valuing that these stages are learning opportunities that can contribute to establishing healthy habits from childhood, thus favoring the care of the mother and child throughout life [30,32].

## 5. Conclusions

In summary, with the limitations of the present cross-sectional study, it was observed that only a small minority of early postpartum had good knowledge about oral health prevention in infants aged 0 to 2 years. In addition, younger and less educated early postpartum women were at greater risk of having little knowledge on this topic. Not having basic education was the main risk factor identified. It is advisable to train professionals who work with pregnant and postpartum women to develop promotion and prevention strategies during pre- and postnatal care in an interdisciplinary manner with the dentist, since this can favor the consolidation of healthy habits in their children from infancy.

## Figures and Tables

**Table 1:** Sociodemographic characteristics of early postpartum women belonging to the gynecology service of a Peruvian public hospital.

Variable	Categories	Frequency	Percentage
Age group	18 to 25 years old	125	41.3
	26 to 35 years old	117	38.6
	36 to 45 years old	61	20.1
Marital status	Unmarried	49	16.2
	Married or cohabiting	254	83.8
Educational level	No education	20	6.6
	Primary School	61	20.1
	Secondary School	127	41.9
	Post-secondary technical education	74	24.4
	University	21	6.9
Number of children	1 child	74	24.4
	2 children	144	47.5
	3 children or more	85	28.1
Monthly family income	125 to 250 dollars	140	46.2
	251 to 400 dollars	120	39.6
	401 to 600 dollars	43	14.2
Having a dentist as a family member	Yes	34	11.2
	No	269	88.8
	**Mean**	**SD**	
Age	28.1	6.4	

SD: Standard deviation.

**Table 2:** Knowledge of early postpartum women about feeding habits in infants aged 0 to 2 years old of age, associated with sociodemographic factors.

Questions	Incorrect	Correct	Age Group	Marital Status	Educational Level	Number of Children	Monthly Family Income	Dentist as a Family Member
	f (%)	f (%)	*p*	*p*	*p*	*p*	*p*	*p*
**Q1.** Until what age should your child be exclusively breastfed?	137 (45.2)	166 (54.8)	0.077	0.372	<0.001 *	0.987	<0.001 *	0.616
**Q2.** Why is breastfeeding important for your child’s mouth?	165 (54.5)	138 (45.5)	<0.001 *	0.096	0.003 *	0.635	<0.001 *	0.006 *
**Q3.** What food is most beneficial for your baby?	92 (30.4)	211 (69.6)	0.439	0.967	0.005 *	0.038 *	0.003 *	0.600
**Q4.** What type of food should be supplemented with breast milk after 6 months of age?	16 (5.3)	287 (94.7)	0.753	0.448	0.770	0.212	0.331	0.810
**Q5.** What is the effect of using a feeding bottle with sweetened milk to put your child to sleep?	184 (60.7)	119 (39.3)	0.040 *	0.094	<0.001 *	0.434	<0.001 *	0.083
**Q6.** From what age is it advisable to give your child liquids complementary to breast milk and how would you give them?	181 (59.7)	122 (40.3)	0.982	0.931	0.763	0.728	0.516	0.035 *

* Based on Pearson’s Chi-square, with Yates correction for expected frequencies less than 5, *p* < 0.05 (significant association).

**Table 3:** Knowledge of early postpartum women about oral hygiene in infants aged 0 to 2 years old, associated with sociodemographic factors.

Questions	Incorrect	Correct	Age Group	Marital Status	Educational Level	Number of Children	Monthly Family Income	Dentist as a Family Member
	f (%)	f (%)	*p*	*p*	*p*	*p*	*p*	*p*
**Q7.** From what age should you start cleaning your child’s mouth?	182 (60.1)	121 (39.9)	0.367	0.256	0.228	0.835	0.427	0.184
**Q8.** At what time of the day should you clean your child’s mouth?	172 (56.8)	131 (43.2)	0.003 *	0.491	<0.001 *	0.002 *	0.032 *	0.797
**Q9.** At what age should you start brushing your child’s teeth with toothpaste?	229 (75.6)	74 (24.4)	0.956	0.004 *	0.003	0.747	0.226	0.472
**Q10.** What actions are part of your child’s oral hygiene?	108 (35.6)	195 (64.4)	0.398	0.071	0.206	0.016 *	0.497	0.738
**Q11.** How should you brush your child’s teeth?	123 (40.6)	180 (59.4)	0.002 *	0.010 *	<0.001 *	0.207	<0.001 *	0.504
**Q12.** What action contributes to the transmission of the microorganism that causes dental caries?	44 (14.5)	259 (85.5)	0.015 *	0.201	0.003 *	0.009 *	0.090	0.974

* Based on Pearson’s Chi-square, with Yates correction for expected frequencies less than 5, *p* < 0.05 (significant association).

**Table 4:** Knowledge of early postpartum women about dental care in infants aged 0 to 2 years old, associated with sociodemographic factors.

Questions	Incorrect	Correct	Age Group	Marital Status	Educational Level	Number of Children	Monthly Family Income	Dentist as a Family Member
	f (%)	f (%)	*p*	*p*	*p*	*p*	*p*	*p*
**Q13.** Why would you take your child to the dentist?	163 (53.8)	140 (46.2)	0.073	0.038 *	<0.001 *	0.841	<0.001 *	0.022 *
**Q14.** At what age should your child’s first visit to the dentist be?	250 (82.5)	53 (17.5)	0.351	0.519	0.218	0.203	0.031 *	0.980
**Q15.** What should you do when your child hits his mouth and starts bleeding?	174 (57.4)	129 (42.6)	0.148	0.030 *	0.207	0.405	0.018 *	0.042 *
**Q16.** How should you clean your baby’s mouth when he/she does not have teeth yet?	29 (9.6)	274 (90.4)	0.114	0.005 *	0.446	0.856	0.379	0.641
**Q17.** When do your child’s first milk teeth start to erupt?	158 (52.1)	145 (47.9)	0.033 *	0.863	0.228	0.740	0.280	0.320
**Q18.** How many milk teeth will your child have?	212 (70.0)	91 (30.0)	0.314	0.003 *	<0.001 *	0.310	0.005 *	0.268

* Based on Pearson’s Chi-square, with Yates correction for expected frequencies less than 5, *p* < 0.05 (significant association).

**Table 5:** Association of sociodemographic factors of early postpartum women with knowledge about oral health prevention in infants aged 0 to 2 years old.

Variable	Categories	Feeding Habits		* *p*	Oral Hygiene		* *p*	Dental Care		*p*	Overall		* *p*
		Unacceptable (Poor)	Acceptable (Fair/Good)		Unacceptable	Acceptable		Unacceptable	Acceptable		Unacceptable	Acceptable	
		f (%)	f (%)		f (%)	f (%)		f (%)	f (%)		f (%)	f (%)	
**Age group**	18 to 25 years old	68 (22.4)	57 (18.8)	0.007 *	79 (26.1)	46 (15.2)	0.029 *	100 (33.0)	25 (8.3)	0.003 *	76 (25.1)	49 (16.2)	<0.001 *
	26 to 35 years old	40 (13.2)	77 (25.4)		54 (17.8)	63 (20.8)		70 (23.1)	47 (15.5)		41 (13.5)	76 (25.1)	
	36 to 45 years old	28 (9.2)	33 (10.9)		34 (11.2)	27 (8.9)		43 (14.2)	18 (5.9)		25 (8.3)	36 (11.9)	
**Marital status**	Unmarried	28 (9.2)	21 (6.9)	0.060	36 (11.9)	13 (4.3)	0.005 *	39 (12.9)	10 (3.3)	0.120	33 (10.9)	16 (5.3)	0.002 *
	Married or cohabiting	108 (35.6)	146 (48.2)		131 (43.2)	123 (40.6)		174 (57.4)	80 (26.4)		109 (36.0)	145 (47.9)	
**Educational level**	No education	13 (4.3)	7 (2.3)	<0.001 *	15 (5.0)	5 (1.7)	0.001 *	18 (5.9)	2 (0.7)	0.004 *	14 (4.6)	6 (2.0)	<0.001 *
	Primary school	34 (11.2)	27 (8.9)		40 (13.2)	21 (6.9)		49 (16.2)	12 (4.0)		36 (11.9)	25 (8.3)	
	Secondary school	65 (21.5)	62 (20.5)		73 (24.1)	54 (17.8)		92 (30.4)	35 (11.6)		67 (22.1)	60 (19.8)	
	Post-secondary technical education	21 (6.9)	53 (17.5)		35 (11.6)	39 (12.9)		42 (13.9)	32 (10.6)		22 (7.3)	52 (17.2)	
	University	3 (1.0)	18 (5.9)		4 (1.3)	17 (5.6)		12 (4.0)	9 (3.0)		3 (1.0)	18 (5.9)	
**Number of children**	1 child	33 (10.9)	41 (13.5)	0.673	44 (14.5)	30 (9.9)	0.005 *	55 (18.2)	19 (6.3)	0.680	39 (12.9)	35 (11.6)	0.501
	2 children	68 (22.4)	76 (25.1)		66 (21.8)	78 (25.7)		99 (32.7)	45 (14.9)		64 (21.1)	80 (26.4)	
	3 children or more	35 (11.6)	50 (16.5)		57 (18.8)	28 (9.2)		59 (19.5)	26 (8.6)		39 (12.9)	46 (15.2)	
**Monthly family income**	125 to 250 dollars	80 (26.4)	60 (19.8)	<0.001 *	86 (28.4)	54 (17.8)	0.020 *	109 (36.0)	31 (10.2)	0.012 *	82 (27.1)	58 (19.1)	<0.001 *
	251 to 400 dollars	48 (15.8)	72 (23.8)		65 (21.5)	55 (18.2)		80 (26.4)	40 (13.2)		51 (16.8)	69 (22.8)	
	401 to 600 dollars	8 (2.6)	35 (11.6)		16 (5.3)	27 (8.9)		24 (7.9)	19 (6.3)		9 (3.0)	34 (11.2)	
**Having a dentist as a family member**	Yes	12 (4.0)	22 (7.3)	0.233	18 (5.9)	16 (5.3)	0.787	18 (5.9)	16 (5.3)	0.019 *	13 (4.3)	21 (6.9)	0.285
	No	124 (40.9)	145 (47.9)		149 (49.2)	120 (39.6)		195 (64.4)	74 (24.4)		129 (42.6)	140 (46.2)	

* Based on Pearson’s Chi-square, with Yates correction for expected frequencies less than 5, *p* < 0.05 (significant association).

**Table 6:** Logistic regression model of overall knowledge about oral health prevention in infants aged 0 to 2 years old, according to associated factors.

Variable	Categories	Crude Model				Adjusted Model			
		OR	95% CI		* *p*	OR	95% CI		* *p*
			LL	UL			LL	UL	
**Age group**	18 to 25 years old	2.19	1.00	4.77	0.049 *	2.43	1.26	4.70	0.008 *
	26 to 35 years old	1.05	0.52	2.14	0.896	1.06	0.54	2.09	0.872
	36 to 45 years old	1.00				1.00			
**Marital status**	Unmarried	1.76	0.84	3.72	0.137				
	Married or cohabiting	1.00							
**Educational level**	No education	7.19	1.15	45.01	0.035 *	11.76	2.41	57.43	0.002 *
	Primary school	4.17	0.80	21.85	0.091	6.61	1.72	25.45	0.006 *
	Secondary school	3.60	0.73	17.85	0.116	5.50	1.52	19.89	0.009 *
	Post-secondary technical education	1.62	0.34	7.67	0.544	2.10	0.55	7.99	0.277
	University	1.00				1.00			
**Number of children**	1 child	0.93	0.42	2.06	0.861				
	2 children	0.85	0.45	1.63	0.629				
	3 children or more	1.00							
**Monthly family income**	125 to 250 dollars	1.64	0.54	4.94	0.379				
	251 to 400 dollars	1.60	0.56	4.52	0.379				
	401 to 600 dollars	1.00							
**Having a dentist as a family member**	Yes	0.96	0.41	2.24	0.921				
	No	1.00							

Logit model: All variables were entered in the statistical analysis of the raw multivariate model. Subsequently, the model was adjusted only with the associated factors (* *p* < 0.05), OR = Odds ratio, 95% CI = 95% confidence interval. For the adjusted model about knowledge, the Pseudo R2 = 0.168, *p* < 0.001 (significant for the omnibus test of the model coefficient).

## Data Availability

The data presented in this study are available on request from the corresponding author.

## Supplementary Materials

The following supporting information can be downloaded at: https://www.mdpi.com/article/10.3390/ijerph20031881/s1, Questionnaire.
