# Supplementary material for: Sociodemographic Factors Associated with the Level of Knowledge of Early Postpartum Women about Oral Health Prevention in Infants Aged 0 to 2 Years Old: A Cross-Sectional Study under a Multivariable Analysis
Source: Int J Environ Res Public Health. 2023 Jan 19;20(3):1881. doi: 10.3390/ijerph20031881 (PMC9914894; doi:10.3390/ijerph20031881)
Supplement: Supplementary file 1 [file ijerph-20-01881-s001.zip › ijerph-2133532-supplementary.pdf]

QUESTIONNAIRE

Feeding habits

Q1. Until what age should your child be exclusively breastfed?

a) Up to 2 months old

b) Up to 4 months old

c) Up to 6 months old

d) Up to 8 months old

Q2. Why is breastfeeding important for your child's mouth?

a) Because your child will have whiter teeth

b) Because it will help your child strengthen the bones of the mouth

c) Because your child will learn to breathe through the mouth.

d) Because your child will speak better

Q3. What food is most beneficial for your baby?

a) Chocolate

b) Coffee

c) Milk

d) Industrialized/processed fruit juices

Q4. What type of food should be supplemented with breast milk after 6 months of age?

a) Porridge, fruits and vegetables

b) Cakes and fruits

c) Do not supplement with anything

d) Sweets and juices

Q5. What is the effect of using a feeding bottle with sweetened milk to put your child to sleep?

a) None

b) That your child can sleep well

c) That your child has cavities

d) That your child has a lot of gas

Q6. From what age is it advisable to give your child liquids complementary to breast milk and how would you give them?

a) At 4 months and with feeding bottle

b) At 4 months and with feeding bottle and glass

c) At 6 months and with feeding bottle.

d) At 6 months and with glass or cup

Oral hygiene

Q7. From what age should you start cleaning your child's mouth?

a) From birth

b) When milk teeth begin to appear

c) When all the milk teeth come in

d) When permanent teeth start to come in

Q8. At what time of the day should you clean your child's mouth?

a) Only in the morning

b) Only in the night

c) Before each meal

d) After each meal

Q9. At what age should you start brushing your child's teeth with toothpaste?

a) From 2 years old.

b) From 5 years old

c) From 6 years old

d) From the eruption of the first tooth

Q10. What actions are part of your child's oral hygiene?

a) Use a large brush.

b) Brushing teeth and tongue

c) Apply plenty of toothpaste to the toothbrush.

d) Rinse with water only.

Q11. How should you brush your child's teeth?

a) All teeth and from top to bottom

b) All teeth and in circular form

c) All teeth and horizontally

d) Front teeth from top to bottom and back teeth in a circular shape

Q12. What action contributes to the transmission of the microorganism that causes dental caries?

a) Give a kiss on the cheek

b) Give a kiss on the mouth

c) Give a kiss on the forehead

d) Give a kiss on the hand

Dental care

Q13. Why would you take your child to the dentist?

a) Due to dental pain

b) Due to inflammation of the face

c) For a medication

d) For prevention of oral diseases

Q14. At what age should your child's first visit to the dentist be?

a) From birth

b) At the first year of age

c) At 3 years old

d) At 4 years old

Q15. What should you do when your child hits his mouth and starts bleeding?

a) Wait until the baby stops bleeding

b) Take it to the dentist

c) Apply medication

d) Wash the mouth with water and salt

Q16. How should you clean your baby's mouth when he/she does not have teeth yet?

a) Moistened gauze

b) With toothbrush

c) Only with water

d) Only with gauze

Q17. When do your child's first milk teeth start to erupt?

a) At birth

b) At approximately 6 months of age

c) At approximately one year of age

d) At 2 years of age

Q18. How many milk teeth will your child have?

a) 12 milk teeth

b) 6 milk teeth

c) 10 milk teeth

d) 20 milk teeth
